# Predicting mortality in acute kidney injury patients undergoing continuous renal replacement therapy using a visualization model: A retrospective study

**DOI:** 10.3389/fphys.2022.964312

**Published:** 2022-11-08

**Authors:** Zhenguo Zeng, Kang Zou, Chen Qing, Jiao Wang, Yunliang Tang

**Affiliations:** ^1^ Department of Critical Care Medicine, The First Affiliated Hospital of Nanchang University, Nanchang, China; ^2^ Department of Endocrinology and Metabolism, The First Affiliated Hospital of Nanchang University, Nanchang, China; ^3^ Department of Rehabilitation Medicine, The First Affiliated Hospital of Nanchang University, Nanchang, China

**Keywords:** acute kidney injury, continuous renal replacement therapy, mortality, visualization, web-based calculator

## Abstract

**Background:** Patients with severe acute kidney injury (AKI) require continuous renal replacement therapy (CRRT) when hemodynamically unstable. We aimed to identify prognostic factors and develop a nomogram that could predict mortality in patients with AKI undergoing CRRT.

**Methods:** Data were extracted from the Dryad Digital Repository. We enrolled 1,002 participants and grouped them randomly into training (*n* = 670) and verification (*n* = 332) datasets based on a 2:1 proportion. Based on Cox proportional modeling of the training set, we created a web-based dynamic nomogram to estimate all-cause mortality.

**Results:** The model incorporated phosphate, Charlson comorbidity index, body mass index, mean arterial pressure, levels of creatinine and albumin, and sequential organ failure assessment scores as independent predictive indicators. Model calibration and discrimination were satisfactory. In the training dataset, the area under the curves (AUCs) for estimating the 28-, 56-, and 84-day all-cause mortality were 0.779, 0.780, and 0.787, respectively. The model exhibited excellent calibration and discrimination in the validation dataset, with AUC values of 0.791, 0.778, and 0.806 for estimating 28-, 56-, and 84-day all-cause mortality, respectively. The calibration curves exhibited the consistency of the model between the two cohorts. To visualize the results, we created a web-based calculator.

**Conclusion:** We created a web-based calculator for assessing fatality risk in patients with AKI receiving CRRT, which may help rationalize clinical decision-making and personalized therapy.

## Background

Acute kidney injury (AKI) is a sudden decline in kidney function that includes structural damage and renal function impairment. AKI occurs in more than half of critically ill patients (Hoste et al., 2015) and has been linked to increased morbidity, mortality, and intensive care unit (ICU) duration of stay (Bouchard et al., 2015; Malhotra et al., 2017). Approximately 40% of patients with AKI develop life-threatening renal dysfunction (Bagshaw et al., 2020), including azotemia, hyperkalemia, oliguria, severe acidosis, or chronic anuria, necessitating continuous renal replacement therapy (CRRT). CRRT is a cornerstone in treating AKI in critically ill patients and may offer greater clearance of toxic compounds, acid-base balance, and elimination of inflammatory mediators that might lead to organ damage and dysfunction than other approaches (Hoffmann et al., 1996; Heering et al., 1997; Honore and Joannes-Boyau, 2004; Rimmelé and Kellum, 2012). The best CRRT mode for AKI is unknown, and clinical practice is diverse (Ronco et al., 2015; Bagshaw et al., 2017). Consequently, AKI prognosis is crucial to determining the optimal treatment plan. However, precise prognostic biomarkers or models are not yet available. Therefore, a precise prediction technique is required.

Typical prognostic indicators for AKI include AKI stage, urine output, and comorbidities. Among these variables, the AKI stage is crucial for estimating outcomes. There is a definite linear association between the AKI stage and mortality (Hoste et al., 2006; Palevsky et al., 2008; Bellomo et al., 2009). Previous research has revealed a mortality rate of 60.5% among anuric patients, 52.6% among oliguric patients, and 34.5% among non-oliguric patients (Choi et al., 2015). The predictive significance of comorbidities is also apparent (Goldberg et al., 2005; Tsai et al., 2014; Platon et al., 2016). Moreover, renal survival at 1, 5, and 10 years was remarkably greater in patients without comorbid diseases (Szerlip and Chawla, 2016). In addition, various urine and blood markers have been studied in recent years to establish whether they can accurately estimate renal damage and prognosis before a decrease in urine output or increase in serum creatinine (Parikh et al., 2005). AKI is a diverse disease; hence, these clinical variables alone cannot properly estimate prognosis. Moreover, estimating AKI prognosis based on a single characteristic is generally inaccurate and unreliable. The integration of clinical models with diverse biomarkers increases the accuracy of outcome estimation.

In this study, we created a model utilizing Cox regression to estimate the short-term prognosis of AKI patients based on regularly utilized and easily accessible clinical variables. Univariate and multivariate analyses were conducted to identify risk variables independent of each other. The visualization model was created using a nomogram and web-based calculator, and the estimation performance was assessed based on discrimination, calibration, and clinical value.

## Methods

### Patients

The data were abstracted from the “DATADRYAD” data resource (www.datadryad.org). It is a user-friendly resource. All authors have waived their copyright to the original research data. Thus, we utilized these data for secondary analysis without violating author rights. This study data was shared by Jung et al. and saved in the Dryad Data Resource (https://datadryad.org/stash/dataset/doi:10.5061%2Fdryad.6v0j9) (Jung, Kwon, Park, Jhee, Yun, Kim, Kee, Yoon, Chang, Kang, Park, Yoo, Kang, Han). As all data were abstracted *via* an online data resource, clearance from the institution’s ethical committee was not required.

Between January 2009 and September 2016, Jung et al. performed a population-based longitudinal investigation at Yonsei University Health System, Severance Hospital and National Health Insurance Service Medical Center, Ilsan Hospital. The inclusion criteria were patients who met the Acute Kidney Injury Network criteria for stage 2 or above (>2-fold increase in blood creatinine or urine output <0.5 ml/kg/h for 12 h). Patients were excluded who met the following criteria: age <18 years, pregnancy or lactation, history of CKD or dialysis or CRRT before the study, postrenal obstruction and prior kidney Transplantation. All eligible patients were randomly assigned to one of two groups in a ratio of 2:1 (training and verification sets).

### Data collection

We conducted a secondary assessment using data from the resources mentioned above. We selected 30 promising prediction indicators, including age, myocardial infarction, sex, congestive heart failure, dementia, hypertension, chronic obstructive pulmonary disease, cerebrovascular disease, potassium, phosphate, Charlson comorbidity index (CCI), body mass index (BMI), systolic blood pressure (SBP), diastolic blood pressure (DBP), mean arterial pressure (MAP), mechanical ventilation (MV), peripheral vascular disease, white blood cells, diabetes mellitus, levels of hemoglobin, blood urea nitrogen, creatinine, and albumin, glomerular filtration rate (GFR), acute physiology and chronic health evaluation II (APACHE II), sequential organ failure assessment (SOFA) score, CRRT cause, and CRRT dose. The vital status and duration of follow-up for each patient with AKI were retrieved.

### Selection of predictive variables and development of the prediction model

Cox proportional hazard regression models were used to screen for possible predictive variables and determine their weights based on the training set. Univariate Cox regression analysis was conducted to identify possible variables. The selected predictive variables from the univariate analysis (*p* < 0.05) were then incorporated into a multivariate Cox regression analysis to generate an integrated nomogram *via* a stepwise feature selection approach based on AIC. To enhance clinical utilization, a web-based calculator visualization tool was developed.

### Validation of the prediction model

We assessed the predictive ability of our model for survival using the area under the receiver operating characteristic curve (ROC) curve (AUC) values from the ROC analysis and C-index. The calibration in the training and verification sets was used to evaluate the performance of the novel model. Decision curve analysis (DCA) was used to check the clinical usefulness of the model. These assessments were conducted using training and validation sets.

### Statistical analysis

Continuous data with a normal distribution were reported as mean ± standard deviation, while categorical variables were expressed as percentages. Analyses of the differences between the training and verification sets were performed using chi-square tests for categorical variables and t-tests for continuous variables. Statistical significance was set at *p* < 0.05. Statistical analyses were performed using SPSS software (v24.0) and R software v3.6.2.

## Results

### Clinical characteristics of patients

**FIGURE 1:**
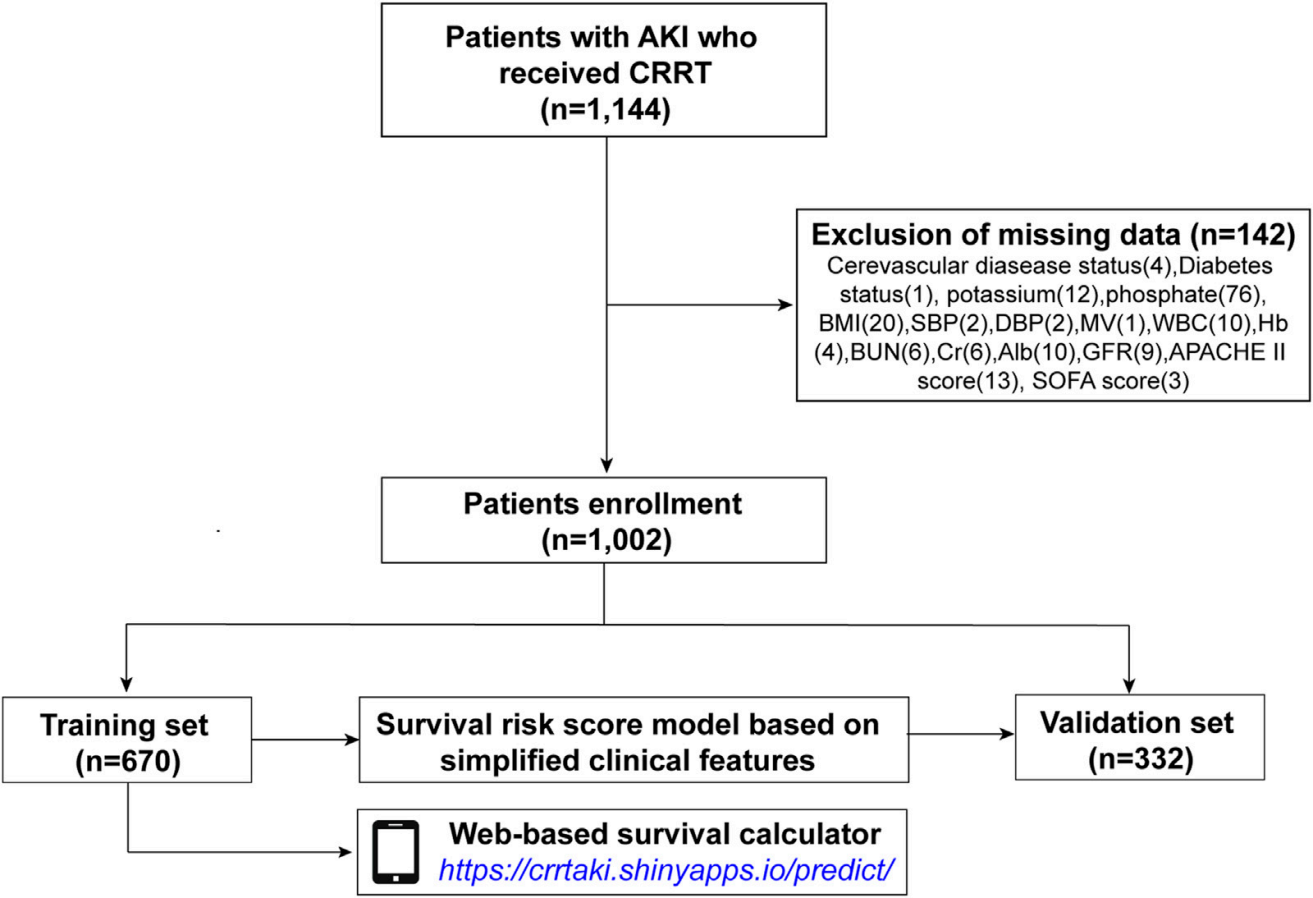
Flow chart of the training and validation cohorts.

This study included 1,002 participants (Figure 1). Patients were randomly divided at a ratio of 2:1 into training (*n* = 670) and validation cohorts (*n* = 332). The demographic and clinical characteristics (before the initiation CRRT) of all participants, and participants in the training and validation sets are presented in Table 1. The mean age of the patients was 63.11 ± 14.46 years, and the study included 623 (62.2%) men and 379 (37.8%) women. The major causes of AKI were sepsis (72%), nephrotoxin (3.3%), ischemia (8.3%), surgery (7.4%), and others (9.1%). Specific data are presented in Table 1.

**TABLE 1:** Baseline demographics and clinical characteristics of patients in training cohort and validation cohort.

Variables	All patients (*n* = 1,002)	Training set (*n* = 670)	Validation set (*n* = 332)	*p*-value
Age, years	63.11 ± 14.46	62.72 ± 14.53	63.91 ± 14.3	0.2201
Gender, n (%)				0.5404
Male	623 (62.2%)	421 (62.8%)	202 (60.8%)	
Female	379 (37.8%)	249 (37.2%)	130 (39.2%)	
Myocardial infarction (%)				0.5946
No	921 (91.9%)	618 (92.2%)	303 (91.3%)	
Yes	81 (8.1%)	52 (7.8%)	29 (8.7%)	
Congestive heart failure (%)				0.7573
No	843 (84.1%))	562 (83.9%)	281 (84.6%)	
Yes	159 (15.9%)	108 (16.1%)	51 (15.4%)	
Cerebrovascular disease (%)				0.4359
No	898 (89.6%)	604 (90.1%)	294 (88.6%)	
Yes	104 (10.4%)	66 (9.9%)	38 (11.4%)	
Peripheral vascular disease (%)				0.1570
No	963 (96.1%)	648 (96.7%)	315 (94.9%)	
Yes	39 (3.9%)	22 (3.3%)	17 (5.1%)	
Dementia (%)				0.2473
No	961 (95.9%)	646 (96.4%)	315 (94.9%)	
Yes	41 (4.1%)	24 (3.6%)	17 (5.1%)	
Diabetes mellitus (%)				0.1723
No	651 (65.0%)	445 (66.4%)	206 (62.0%)	
Yes	351 (35.0%)	225 (33.6%)	126 (38.0%)	
Hypertension (%)				0.7605
No	467 (46.6%)	310 (46.3%)	157 (47.3%)	
Yes	535 (53.4%)	360 (53.7%)	175 (52.7%)	
COPD (%)				0.1021
No	925 (92.3%)	625 (93.3%)	300 (90.4%)	
Yes	77 (7.7%)	45 (6.7%)	32 (9.6%)	
Potassium (mEq/L)	4.71 ± 1.1	4.71 ± 1.11	4.72 ± 1.07	0.8792
Phosphate (mg/dl)	5.74 ± 2.4	5.79 ± 2.49	5.64 ± 2.22	0.3488
Charlson comorbidity index	3.15 ± 2.25	3.16 ± 2.26	3.12 ± 2.22	0.7646
BMI (kg/m^2^)	23.89 ± 4.56	23.91 ± 4.51	23.83 ± 4.67	0.7900
SBP (mmHg)	112.08 ± 20.89	111.9 ± 20.94	112.44 ± 20.8	0.6996
DBP (mmHg)	60.45 ± 14.05	60.37 ± 14.13	60.62 ± 13.91	0.7896
MAP (mmHg)	77.68 ± 14.11	77.88 ± 14.13	77.25 ± 14.09	0.5041
MV (%)				0.5385
No	215 (21.5%)	140 (20.9%)	75 (22.6%)	
Yes	787 (78.5%)	530 (79.1%)	257 (77.4%)	
White blood cell (μl)	13890.47 ± 11172.25	14119.6 ± 11547.87	13428.07 ± 10374.32	0.3567
Hemoglobin (g/dl)	9.63 ± 2.23	9.5 ± 2.21	9.89 ± 2.26	0.0100
BUN (mg/dl)	56.61 ± 30.27	55.08 ± 29.62	59.69 ± 31.35	0.0232
Creatinine (mg/dl)	2.76 ± 1.65	2.75 ± 1.65	2.76 ± 1.67	0.9517
Albumin (g/dl)	2.6 ± 0.58	2.56 ± 0.58	2.66 ± 0.56	0.0090
GFR	31.07 ± 21.58	31.41 ± 22.22	30.4 ± 20.25	0.4850
APACHE II score	27.29 ± 7.98	27.05 ± 8.02	27.78 ± 7.88	0.1732
SOFA score	12.15 ± 3.56	12.18 ± 3.55	12.1 ± 3.58	0.7386
CRRT cause				0.1152
Volume overload (%)	118 (11.8%)	81 (12.1%)	37 (11.1%)	
Metabolic acidosis (%)	216 (21.6%)	155 (23.1%)	61 (18.4%)	
Hyperkalemia (%)	46 (4.6%)	33 (4.9%)	13 (3.9%)	
Uremia (%)	102 (10.2%)	61 (9.1%)	41 (12.3%)	
Oliguria (%)	269 (26.8%)	177 (26.4%)	92 (27.7%)	
Others (%)	251 (25.0%)	163 (24.3%)	88 (26.5%)	
CRRT dose (ml/kg)	36.64 ± 4.7	36.76 ± 4.77	36.4 ± 4.56	0.2582
AKIN stages				0.4236
Stage 2 (%)	251 (25.0%)	173 (25.8%)	78 (23.5%)	
Stage 3 (%)	751 (75.0%)	497 (74.2%)	254 (76.5%)	
AKI cause				0.8023
Sepsis (%)	721 (72.0%)	481 (71.8%)	240 (72.3%)	
Nephrotoxin (%)	33 (3.3%)	24 (3.6%)	9 (2.7%)	
Ischemia (%)	83 (8.3%)	54 (8.1%)	29 (8.7%)	
Surgery (%)	74 (7.4%)	46 (6.9%)	28 (8.4%)	
Others (%)	91 (9.1%)	65 (9.7%)	26 (7.8%)	

COPD, Chronic obstructive pulmonary disease; CCI, Charlson comorbidity index; BMI, Body mass index; MV, Mechanical Ventilation; AKIN, Acute kidney injury criteria; CRRT, Continuous renal replacement therapy; GFR, Glomerular filtration rate; SOFA, Sequential Organ Failure Assessment Score; APACHE II, Acute Physiology and Chronic Health Evaluation II, SBP, Systolic blood pressure; DBP, Diastolic blood pressure; MAP, Mean arterial pressure.

### Univariate and multivariate analyses in the training cohort

Univariate and multivariate analyses were performed to screen independent prognostic factors to build the model. Univariate Cox regression analysis indicated that diabetes mellitus, hypertension, phosphate, CCI, BMI, SBP, DBP, MAP, MV, levels of creatinine and albumin, GFR, and APACHE II and SOFA scores correlated with survival time. Multivariate Cox regression analysis showed that phosphate, CCI, BMI, MAP, levels of creatinine and albumin, and SOFA score were independent prognostic factors for patients with AKI (Supplementary Table S1).

### Prediction model construction

**FIGURE 2:**
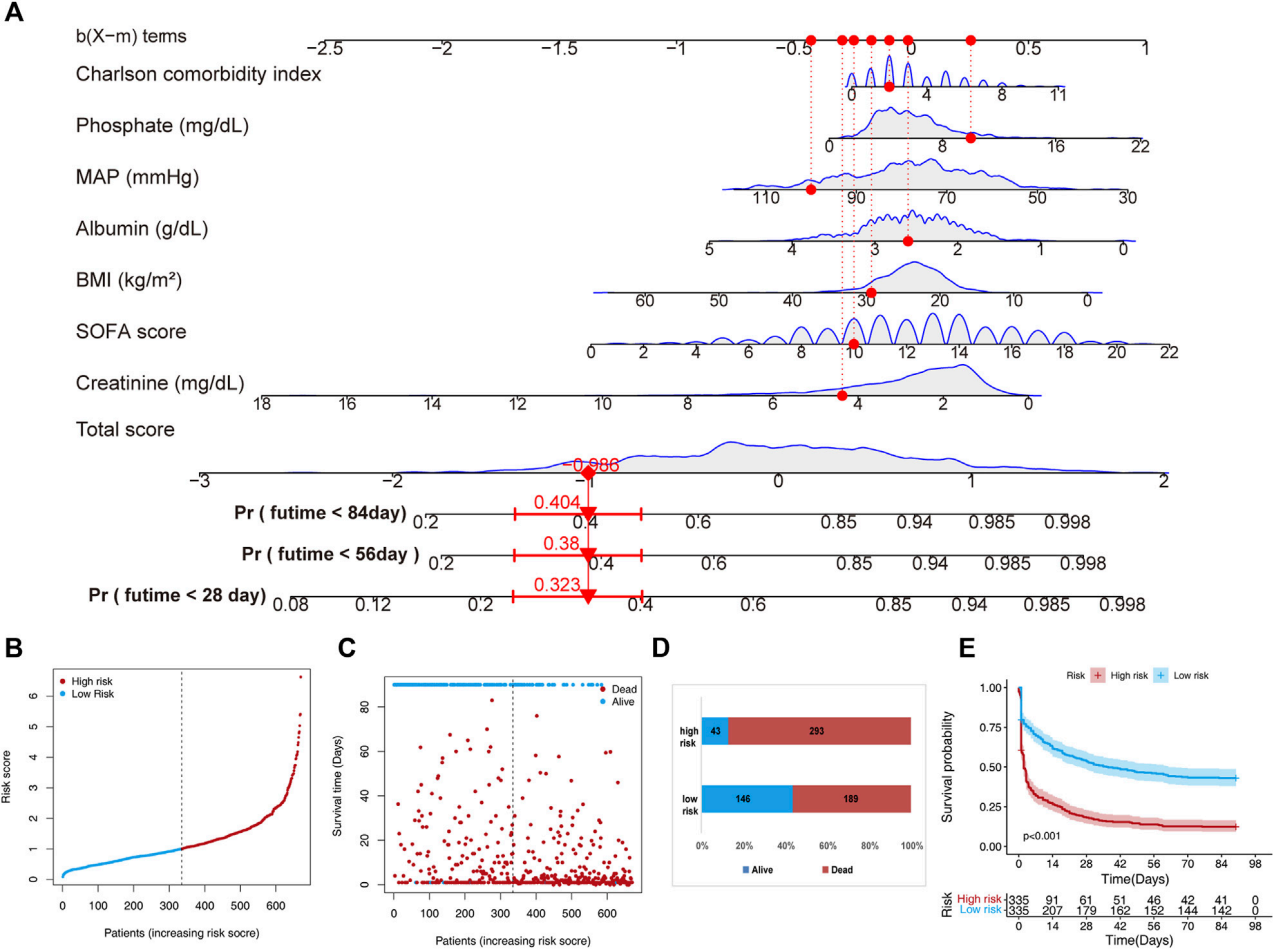
A nomogram developed for predicting short-term mortality based on the training set. **(A)** The nomogram is based on the seven factors determined using Cox’s assessment of risks. **(B)** The distribution of risk scores is derived from the nomogram scoring approach. **(C)** Distribution of participants in the low and high score categories based on their survival status. **(D)** Comparison of survival risk between the two groups. **(E)** Overall survival curves separated by groups with low and high scores.

Through multivariate Cox regression analysis screening, the final prediction model established included phosphate, CCI, BMI, MAP, levels of creatinine and albumin, and SOFA scores as predictors. The prediction model was presented in a nomogram, which was used to quantitatively predict the 28-, 56-, and 84- day overall survival probability (Figure 2A). A total score could be easily obtained by adding each single score of the selected variables, and then projecting the total score to the bottom scale can estimate the probabilities of 28-, 56-, and 84-day survival for each individual patient. Next, according to this prediction model, the 670 patients in the training set were stratified into low-(n = 335) and high-risk (*n* = 335) groups. The risk score distribution in the training set is shown in Figure 2B, and the distribution of survival status ranked by the risk score is presented in Figure 2C. Individuals were at greater risk of mortality with increasing risk scores (Figure 2D). Moreover, we utilized survival analysis to compare high-and low-risk patients, and the results showed that the high-risk group exhibited a significantly poorer survival rate than the low-risk group (Figure 2E).

### Development of a web-based calculator

**FIGURE 3:**
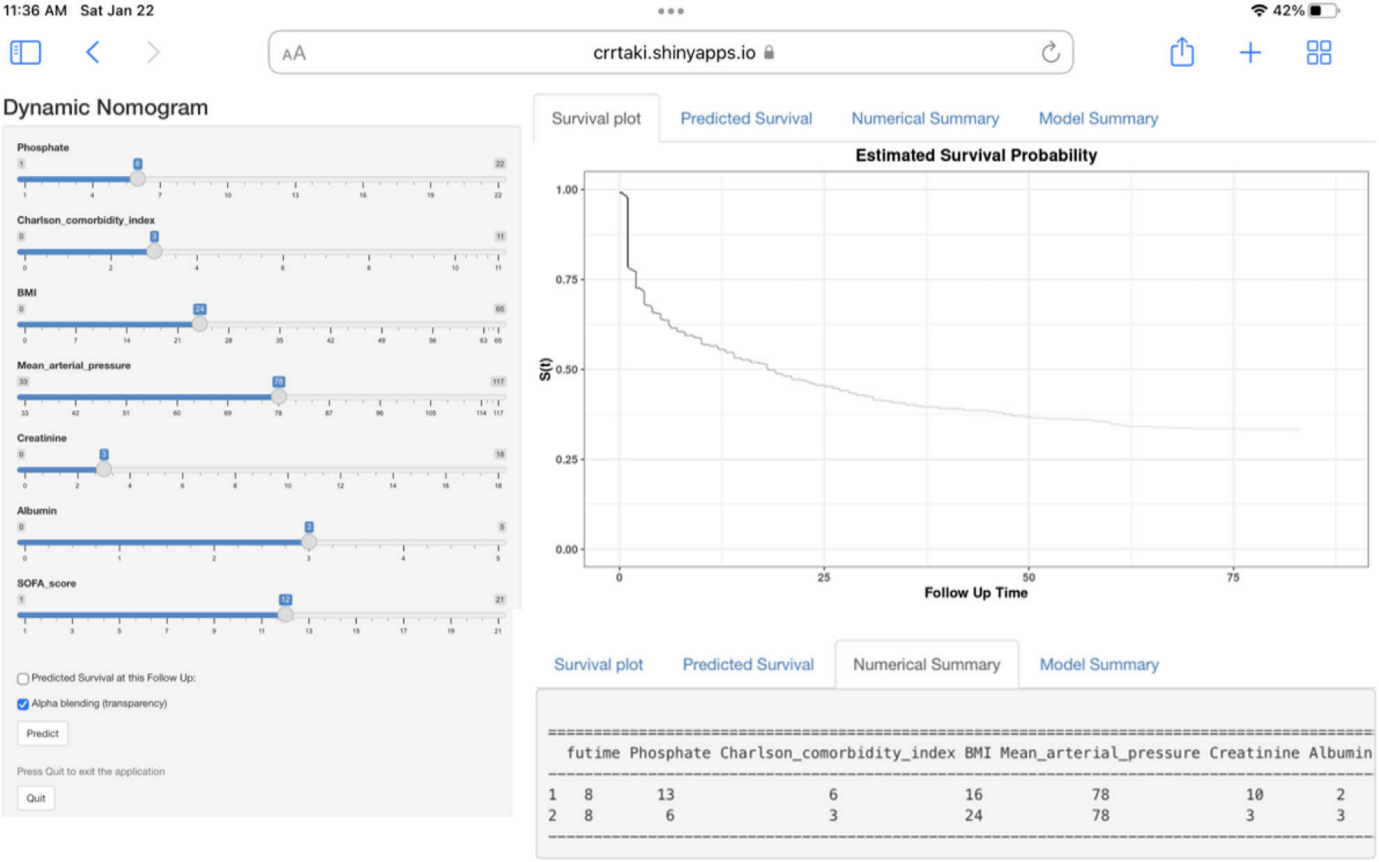
Web-based dynamic nomogram for estimating the probability of short-term mortality. By entering the specifics of the acute kidney injury participant receiving continuous renal replacement therapy into the web-based program, we determined the participant’s short-term all-cause death probability (Entering Interface: This interface allows you to enter participant-specific information. Short-term all-cause death probability, as depicted graphically in part, https://crrtaki.shinyapps.io/predict/).

We built an easy-to-use web-based resource (https://crrtaki.shinyapps.io/predict/) for clinical use and visualization of the prediction model to estimate the course of AKI based on the nomogram (Figure 3). The estimated probabilities of disease progression may be derived by drawing a perpendicular line from the total point axis to the outcome axis.

### Model performance in the training set

**FIGURE 4:**
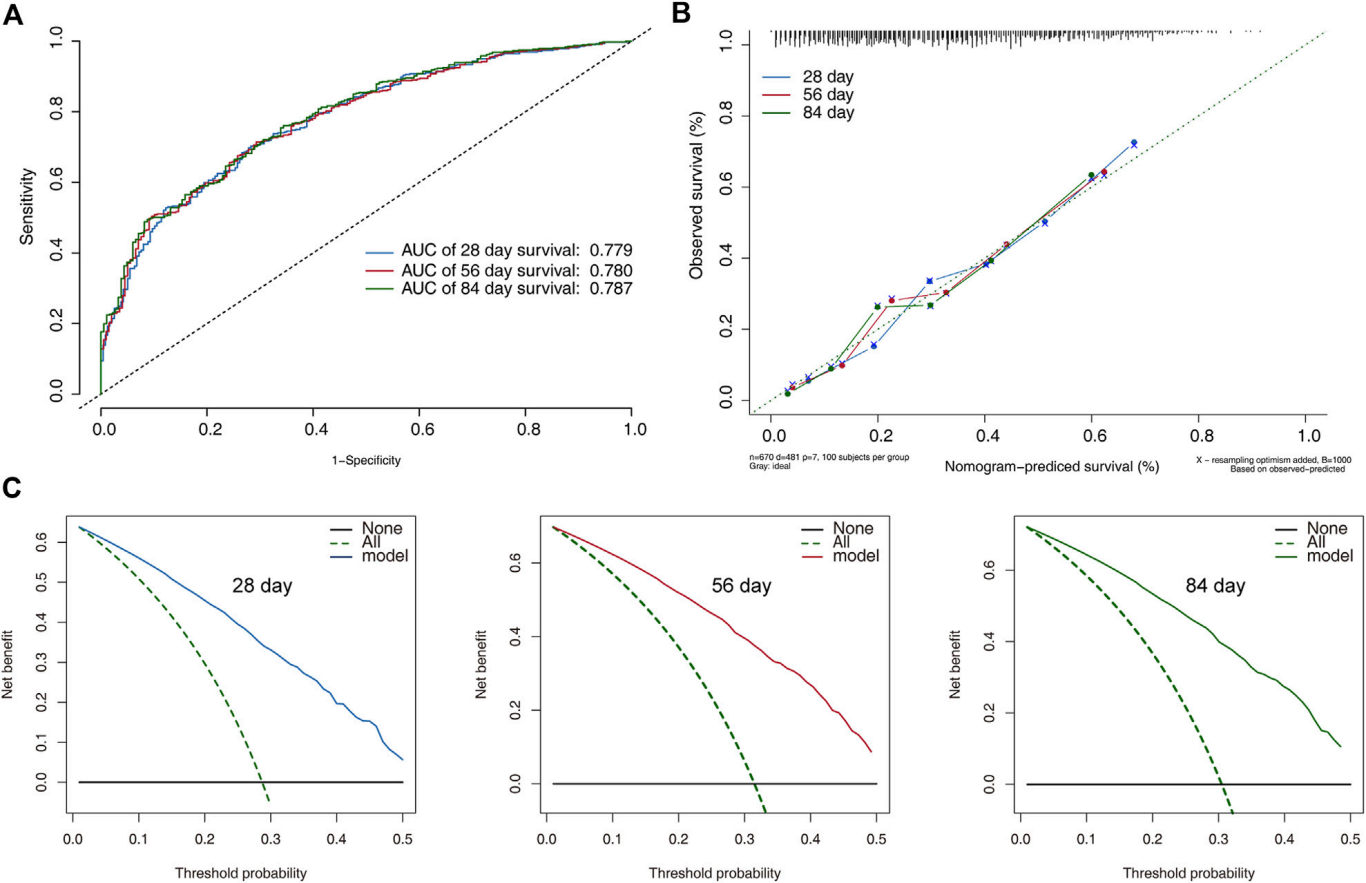
Model performance and discrimination in the training set. **(A)** Receiver operating characteristic curves for model-based prediction of overall survival. **(B)** Calibration curves for the monogram in the cohort, where the *x* and *y* axes show the projected probability and the observed probability of all-cause mortality. **(C)** The decision curve analysis of the training cohort’s 28-, 56-, and 84-day survival nomogram. The area between the “no treatment line” (black line) and the “all treatment line” (light gray line) on the model’s curve represents its clinical value.

In the training set, the discrimination power of the model was assessed using C-index values and ROC curves. The C-index of the model was 0.69. The AUC for the 28-day, 56-day, and 84-day survival predictions were 0.779, 0.780, and 0.787 (Figure 4A), indicating the model’s efficacy in estimating prognosis. The calibration plots derived from the training set demonstrated that the model successfully estimated the 28-, 56-, and 84-day survival rates (Figure 4B). The DCA findings also showed that the model might assist clinicians in optimizing their clinical decision-making processes (Figures 4C–E).

### Model performance in the validation set

The risk scores and survival status distributions (Supplementary Figures S1A,B) displayed a comparable pattern across the high-and low-risk groups in the verification set. According to survival analysis, low-risk patients had a better prognosis than high-risk patients (Supplementary Figure S1C). The 28-, 56-, and 84-day AUCs were 0.791, 0.778, and 0.806 (Supplementary Figure S2A). The training set calibration plots indicated that the model could reliably predict 28-, 56-, and 84-day survival rates (Supplementary Figure S2B). DCA findings showed that the model might assist clinicians in making better clinical judgments (Supplementary Figures S2C).

## Discussion

AKI is a prevalent disease with a rising frequency among hospital and ICU patients (Romagnoli et al., 2018; Pistolesi et al., 2019). Therefore, the risk of classifying patients and estimating their prognosis is vital to properly educate the patient and family, utilize healthcare resources effectively, provide treatment interventions that could improve outcomes, and develop treatments aimed at altering markers related to these dismal outcomes.

In this retrospective investigation, risk variables for short-term prognosis were assessed using Cox regression analysis, and an estimation model of predictive risk was created using clinical data and demographic information of individuals with AKI. According to our study findings, phosphate, CCI, BMI, MAP, levels of creatinine and albumin, and SOFA score were independent risk factors for AKI. Based on these risk factors, a model was generated to estimate the short-term survival of patients with AKI. The nomogram could be utilized as a cost-effective tool to estimate the prognosis of AKI and aid in clinical decision-making.

A nomogram is a simple tool that can estimate risk by creating a visible picture; it is frequently used in clinical practice (Tang et al., 2020; Wang et al., 2020; Tang et al., 2021). A nomogram that incorporates several key features is a simple and easy-to-use tool that clinicians may use to diagnose and estimate various patient groups’ prognoses. In the current study, we created a nomogram based on independent predictive risk variables to estimate the survival of patients with AKI undergoing CRRT. In addition to the classic nomogram, we created a dynamic nomogram that could estimate patient prognosis using a simple web page operation. Unlike previous nomograms that calculated an estimate, the dynamic nomogram may deliver exact results.

Clinical research has sought to develop prognostic indicators and models for estimating AKI outcomes. Regrettably, most of these predictors and models performed poorly in terms of discrimination. AKI stage is crucial in determining the outcomes; AKI stage and mortality have a linear relationship. AKI prognostic predictors are crude and only indicate the likelihood of survival. For instance, APACHE II and SOFA scores are commonly used to estimate the clinical prognosis of patients in the ICU. However, previous research has revealed that APACHE II and SOFA scores are poor predictors of prognosis in individuals with AKI (Uchino et al., 2005).

In recent years, urine and blood biomarkers have been studied to assess whether they can accurately predict renal damage before a drop in urine output or an increase in serum creatinine levels. Biomarkers have also been explored to determine whether they can be used to stratify patients into mortality risk categories. Parikh et al. established that urine interleukin-18 is an independent predictor of mortality in patients with acute renal injury in the intensive care unit. A urine interleukin-18 concentration >100 pg/ml increases the risk of death by 1.6-fold (Parikh et al., 2005). In a group of critically ill patients admitted to the ICU, tissue inhibitors of metalloproteinase-2 and insulin-like growth factor-binding protein-7 were useful in estimating prognosis (Koyner et al., 2015). Elevated plasma levels of miR-210 were an independent predictor of death by Lorenzen et al. (2011) (hazard ratio 1.692).

Previous research on AKI prediction models has mostly focused on specific patient groups, including AKI after cardiac surgery, AKI following noncardiac surgery, septic AKI, tumor-related AKI, and critical AKI (Park et al., 2019; Zhou et al., 2019; Chen et al., 2020; Li et al., 2020; Fan et al., 2021; Hu et al., 2021; Huang et al., 2021; Zhao et al., 2021). James et al. developed an estimation progression model to advanced chronic kidney disease (CKD) after discharge in individuals with AKI, and their data suggested that older age, female sex, higher baseline creatinine levels, elevated serum proteinuria, more severe AKI, and elevated creatinine at discharge were associated with an increased incidence of progression to advanced CKD (James et al., 2017). Nevertheless, few investigations have created or validated estimation models for all-cause mortality in patients with AKI receiving CRRT. To our knowledge, this is the first study to build a predictive model for the survival rate in AKI patients receiving CRRT, which can assist in identifying risk factors for poor prognosis in critically ill patients with AKI early on and improve the short-term prognosis by prompt treatment.

espite its relatively large sample size, this study has four limitations. First, the retrospective analysis was based on American public databases, which may have resulted in several inherent or selected biases. Second, although the model performed well in the training and validation sets, multicenter clinical application is required to determine the external value of the model. Third, the present investigation may have overlooked a few possible risk variables, including the albumin-to-creatinine ratio, medications, and other therapeutic measures.

## Conclusion

We constructed and validated a model incorporating seven clinical characteristics (phosphate, CCI, BMI, MAP, levels of creatinine, levels of albumin, and SOFA score) to predict mortality in patients with AKI undergoing CRRT. This model may be a reliable tool for determining AKI treatment strategies and potential outcomes. The new, user-friendly nomogram developed in this study may accurately estimate the prognosis of AKI patients.

## Data Availability

The original contributions presented in the study are included in the article/Supplementary Material; further inquiries can be directed to the corresponding authors.

## Abbreviations

AKI, acute kidney injury; AUC, area under the curve; APACHE II, acute physiology and chronic health evaluation II; BMI, body mass index; CRRT, continuous renal replacement therapy; CKD, chronic kidney disease; CCI, Charlson comorbidity index; DBP, diastolic blood pressure; DCA, decision curve analysis; GFR, glomerular filtration rate; ICU, intensive care unit; MAP, mean arterial pressure; MV, mechanical ventilation; ROC, receiver operating characteristic; SOFA, Sequential Organ Failure Assessment; SBP, systolic blood pressure.
