# Supplementary material for: Predicting mortality in acute kidney injury patients undergoing continuous renal replacement therapy using a visualization model: A retrospective study
Source: Front Physiol. 2022 Nov 8;13:964312. doi: 10.3389/fphys.2022.964312 (PMC9679412; doi:10.3389/fphys.2022.964312)
Supplement: Supplementary file 1 [file DataSheet2.PDF]

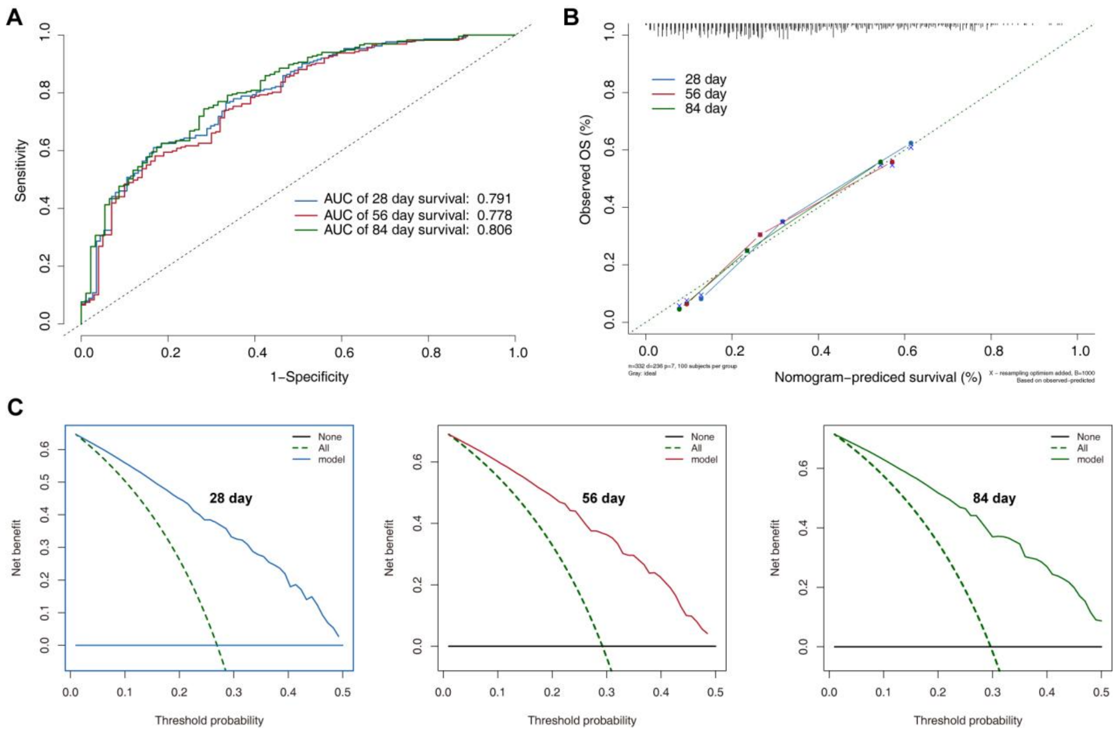

Figure S2: Model performance and discrimination in the verification set. (A) Receiver operating characteristic curves for model-based estimation of overall survival. (B) Calibration curves for the monogram in the cohort, where the x and y axes show the projected probability and the observed probability of all-cause mortality. (C) The decision curve analysis of the nomogram for 28-, 56-, and 84-day survival in the training cohort. The area between the "no treatment line" (black line) and the "all treatment line" (light gray line) on the model's curve represents its clinical value.
