# Supplementary material for: Predicting mortality in acute kidney injury patients undergoing continuous renal replacement therapy using a visualization model: A retrospective study
Source: Front Physiol. 2022 Nov 8;13:964312. doi: 10.3389/fphys.2022.964312 (PMC9679412; doi:10.3389/fphys.2022.964312)
Supplement: Supplementary file 2 [file Table1.docx]

| Table S1. Univariate and multivariable Cox hazards analysis in the training cohort. | | | | | |
| --- | --- | --- | --- | --- | --- |
| Variables | Univariate |  |  | Multivariate |  |
|  | HR (95% CI) | P-value |  | HR (95% CI) | P-value |
| Age, years | 1.003 (0.996-1.009) | 0.417 |  |  |  |
| Gender |  |  |  |  |  |
| Male | Ref. | - |  |  |  |
| Female | 1.026 (0.853-1.234) | 0.786 |  |  |  |
| Myocardial infarction |  |  |  |  |  |
| No | Ref. | - |  |  |  |
| Yes | 0.922 (0.657-1.295) | 0.640 |  |  |  |
| Congestive heart failure |  |  |  |  |  |
| No | Ref. | - |  |  |  |
| Yes | 0.941 (0.739-1.199) | 0.624 |  |  |  |
| Cerebrovascular disease |  |  |  |  |  |
| No | Ref. | - |  |  |  |
| Yes | 0.971 (0.716-1.316) | 0.849 |  |  |  |
| Peripheral vascular disease |  |  |  |  |  |
| No |  |  |  |  |  |
| Yes | 1.134 (0.689-1.867) | 0.620 |  |  |  |
| Dementia |  |  |  |  |  |
| No | Ref. | - |  |  |  |
| Yes | 0.608 (0.343-1.078) | 0.089 |  |  |  |
| Diabetes mellitus |  |  |  |  |  |
| No | Ref. | - |  |  |  |
| Yes | 0.808 (0.666-0.981) | 0.032 |  | 0.955 (0.775-1.177) | 0.667 |
| Hypertension |  |  |  |  |  |
| No | Ref. | - |  |  |  |
| Yes | 0.726 (0.607-0.868) | 0.000 |  | 0.959 (0.785-1.171) | 0.959 |
| COPD |  |  |  |  |  |
| No | Ref. | - |  |  |  |
| Yes | 0.783 (0.534-1.146) | 0.208 |  |  |  |
| Potassium(mEq/L) | 1.009 (0.929-1.095) | 0.837 |  |  |  |
| Phosphate(mg/dL) | 1.051 (1.017-1.087) | 0.003 |  | 1.052 (1.013-1.093) | 0.009 |
| Charlson comorbidity index | 1.076 (1.036-1.117) | 0.000 |  | 1.081 (1.040-1.125) | 0.000 |
| BMI (kg/m^2^) | 0.975 (0.955-0.995) | 0.016 |  | 0.970 (0.949-0.992) | 0.007 |
| SBP (mmHg) | 0.988 (0.983-0.992) | 0.000 |  | 0.996 (0.989-1.003) | 0.228 |
| DBP(mmHg) | 0.990 (0.983-0.996) | 0.002 |  | 1.008 (0.995-1.021) | 0.219 |
| MAP(mmHg) | 0.983 (0.977-0.990) | 0.000 |  | 0.980 (0.965-0.996) | 0.013 |
| MV |  |  |  |  |  |
| No | Ref. | - |  |  |  |
| Yes | 1.471 (1.162-1.862) | 0.001 |  | 0.768 (0.577-1.022) | 0.070 |
| White blood cell (μL) | 1.000 (1.000-1.000) | 0.076 |  |  |  |
| Hemoglobin (g/dL) | 0.962 (0.922-1.004) | 0.077 |  |  |  |
| BUN(mg/dL) | 1.001(0.998-1.004) | 0.465 |  |  |  |
| Creatinine (mg/dL) | 0.908 (0.855-0.964) | 0.002 |  | 0.838 (0.769-0.912) | 0.000 |
| Albumin(g/dL) | 0.712 (0.612-0.827) | 0.000 |  | 0.730 (0.625-0.854) | 0.000 |
| GFR | 1.004 (1.001-1.007) | 0.023 |  | 1.001 (0.996-1.006) | 0.750 |
| APACHE II score | 1.024 (1.013-1.036) | 0.000 |  | 1.010 (0.997-1.024) | 0.126 |
| SOFA score | 1.118 (1.089-1.148) | 0.000 |  | 1.119 (1.084-1.155) | 0.000 |
| CRRT cause |  |  |  |  |  |
| Volume overload | Ref. | - |  |  |  |
| Metabolic acidosis | 1.355 (0.977-1.878) | 0.068 |  |  |  |
| Hyperkalemia | 1.473 (0.925-2.346) | 0.102 |  |  |  |
| Uremia | 1.102 (0.734-1.656) | 0.638 |  |  |  |
| Oliguria | 1.052 (0.759-1.458) | 0.760 |  |  |  |
| Others | 1.317 (0.953-1.821) | 0.096 |  |  |  |
| CRRT dose (ml/kg) | 1.013 (0.995-1.031) | 0.157 |  |  |  |
| AKIN stages |  |  |  |  |  |
| Stage 2 | Ref. | - |  |  |  |
| Stage 3 | 0.999 (0.813-1.228) | 0.993 |  |  |  |
| AKI cause |  |  |  |  |  |
| Sepsis | Ref. | - |  |  |  |
| Nephrotoxin | 1.043 (0.641-1.697) | 0.867 |  |  |  |
| Ischemia | 1.136 (0.822-1.570) | 0.440 |  |  |  |
| Surgery | 0.875 (0.609-1.258) | 0.472 |  |  |  |
| Others | 0.941 (0.690-1.284) | 0.703 |  |  |  |
| COPD, Chronic obstructive pulmonary disease; CCI, Charlson comorbidity index; BMI, Body mass index; MV, Mechanical Ventilation; AKIN, Acute kidney injury criteria; CRRT, Continuous renal replacement therapy; GFR, Glomerular filtration rate; SOFA, Sequential Organ Failure Assessment Score; APACHE II, Acute Physiology and Chronic Health Evaluation II, SBP, Systolic blood pressure; DBP, Diastolic blood pressure; MAP, Mean arterial pressure. | | | | | |
