# Supplementary material for: Predicting mortality in acute kidney injury patients undergoing continuous renal replacement therapy using a visualization model: A retrospective study
Source: Front Physiol. 2022 Nov 8;13:964312. doi: 10.3389/fphys.2022.964312 (PMC9679412; doi:10.3389/fphys.2022.964312)
Supplement: Supplementary file 3 [file DataSheet1.PDF]

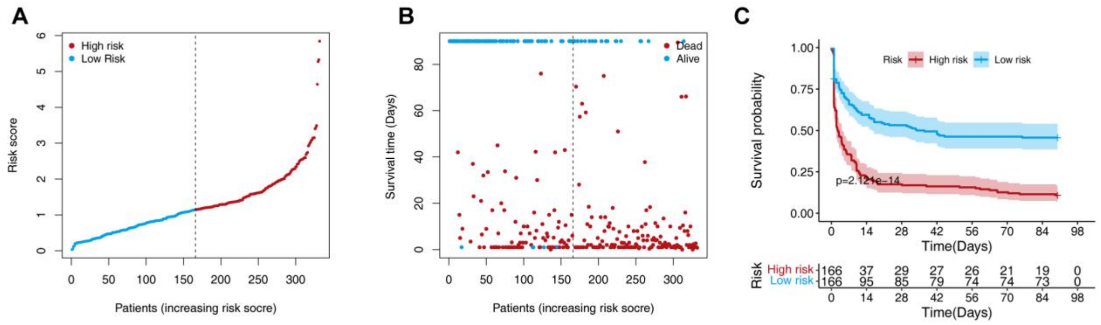

Figure S1: Nomogram verification in the validation set. (A) The distribution of risk scores is derived from the nomogram scoring system. (B) Distribution of patients into the low-and high-score groups based on the survival status. (C) Survival curves separated by groups with low and high scores.
